# Ligand-Bound GeneSwitch Causes Developmental Aberrations in *Drosophila* that Are Alleviated by the Alternative Oxidase

**DOI:** 10.1534/g3.116.030882

**Published:** 2016-07-12

**Authors:** Ana Andjelković, Kia K. Kemppainen, Howard T. Jacobs

**Affiliations:** * BioMediTech, FI-33520, University of Tampere, Finland; † Tampere University Hospital, FI-33014, University of Tampere, Finland; ‡ Institute of Biotechnology, FI-00014, University of Helsinki, Finland

**Keywords:** inducible transgenes, nuclear receptor, *Drosophila*, cleft thorax, notched wings

## Abstract

Culture of *Drosophila* expressing the steroid-dependent GeneSwitch transcriptional activator under the control of the ubiquitous α*-tubulin* promoter was found to produce extensive pupal lethality, as well as a range of dysmorphic adult phenotypes, in the presence of high concentrations of the inducing drug RU486. Prominent among these was cleft thorax, seen previously in flies bearing mutant alleles of the nuclear receptor Ultraspiracle and many other mutants, as well as notched wings, leg malformations, and bristle abnormalities. Neither the α*-tubulin*-GeneSwitch driver nor the inducing drug on their own produced any of these effects. A second GeneSwitch driver, under the control of the *daughterless* promoter, which gave much lower and more tissue-restricted transgene expression, exhibited only mild bristle abnormalities in the presence of high levels of RU486. Coexpression of the alternative oxidase (AOX) from *Ciona intestinalis* produced a substantial shift in the developmental outcome toward a wild-type phenotype, which was dependent on the AOX expression level. Neither an enzymatically inactivated variant of AOX, nor GFP, or the alternative NADH dehydrogenase Ndi1 from yeast gave any such rescue. Users of the GeneSwitch system should be aware of the potential confounding effects of its application in developmental studies.

The GeneSwitch (GS) system is commonly used to activate transgenes in *Drosophila* in a graded fashion. GS comprises a modified form of the yeast transcriptional activator Gal4, which is covalently linked to the hormone-binding fragment of the progesterone receptor, rendering its transcriptional activity dependent on an exogenously supplied progesterone analog, RU486 or mifepristone (Osterwalder *et al.* 2001). Any transgene governed by the UAS promoter element, rendering it Gal4-responsive, may be induced by the combination of GS and RU486 in a dose-dependent manner. Depending on the promoter to which GS is itself combined, plus its insertion site in the fly genome, drug-inducible transgene expression can be achieved in a wide variety of developmental patterns, cell-types, and overall strengths. Thus, the widely used α*-tubulin-GS* (*tubGS*) and *actin5C-GS* drivers confer ubiquitous, RU486-dependent transgene expression when crossed to lines bearing a UAS-governed transgene. Tissue-specific drivers such as the neuron-specific *elav-GS* enable transgene expression in just one tissue, but again at a level and timing that can be manipulated over a wide range. The use of this system is predicated on the assumption that the expression of GeneSwitch and exposure to RU486 do not themselves produce measurable effects on fly physiology and development, which is supported by controls in many studies.

Our laboratory has made use of this system, for example to express, in *Drosophila*, foreign transgenes coding for nonproton-motive alternative respiratory chain enzymes derived from simpler eukaryotes, such as the alternative oxidase (AOX) from *Ciona intestinalis* (Fernandez-Ayala *et al.* 2009; Kemppainen *et al.* 2014a). When supplied to adult *Drosophila* bearing both *tubGS* and a *UAS-AOX* transgene, RU486 produced dose-dependent transgene expression that saturated at drug concentrations (in fly food) of 100–200 μM (Kemppainen *et al.* 2014a). However, when supplied throughout development, RU486 concentrations two orders of magnitude lower were sufficient to induce maximal expression (Fernandez-Ayala *et al.* 2009). The precise reasons for this discrepancy in required dose are unclear, although early larvae, which are very rapidly growing (Church and Robertson 1966; Watts *et al.* 2006), must absorb larger amounts of drugs added to fly food than adults, which do not grow at all and even lose weight during early adult life (Fernandez-Ayala *et al.* 2009).

In this study, we addressed the issue of what happens to development when larvae expressing GeneSwitch drivers (but no other transgene) are exposed to RU486 concentrations in excess of those sufficient to produce maximal transgene expression. We detected a variety of developmental abnormalities dependent on driver expression and drug dose. Surprisingly, expression of AOX, but not other transgenes such as GFP or the yeast alternative NADH dehydrogenase Ndi1, mitigated these effects.

## Materials and Methods

### Drosophila stocks and maintenance

Wild-type (Oregon R), standard transgenic host strains *w^1118^* and *w^DAH^* (Dahomey) and the UAS-GFP (Stinger) line (insertion on chromosome 2) were obtained from stock centers. The *tubGS* driver line with insertion on chromosome 3 (Sykiotis and Bohmann 2008) was a kind gift from Dr Scott Pletcher (University of Michigan). The *daughterless-GS* (*daGS*) line (Tricoire *et al.* 2009) was a kind gift from Dr Alberto Sanz (Newcastle University, UK). AOX and Ndi1 transgenic flies [lines *UAS-AOX^F6^*, *UAS-AOX^F24^*, *tub-AOX^7^*, *tub-AOX^35^ tub-AOX^50^*, *UAS-AOX^7.1^* (targeted insertion on chromosome 3) *UAS-AOX^mut^* (denoted previously as *UAS-AOX^4.1^*, targeted insertion on chromosome 3), and *UAS-Ndi1^B20^*] were as described previously (Fernandez-Ayala *et al.* 2009; Sanz *et al.* 2010b; Kemppainen *et al.* 2014b; Andjelković *et al.* 2015). Flies were maintained in standard high-sugar medium (Fernandez-Ayala *et al.* 2009) at 25°, on a 12 hr light/dark cycle. Where indicated, medium was supplemented with RU486 (Mifepristone, Sigma) at the concentrations indicated in figures and legends.

### Eclosion and phenotypic assays

Crosses were conducted in a minimum of three, usually four to five replicates, as described previously (Toivonen *et al.* 2001; Kemppainen *et al.* 2009). Either the number of flies eclosing or the percentage of pupae that successfully eclosed in individual vials were recorded in different experiments (see figures and legends). The proportion of the eclosed progeny falling into different phenotypic classes was scored by microscopy. Cleft thorax, where subclassified, was scored as mild or severe (heminota clearly separated), with the mildest abnormality, malformed scutellum, scored separately in some experiments. Wing phenotypes were scored as normal or notched, the latter ranging from single notches to grossly malformed wings that in some cases did not inflate properly. Flies showing any of the bristle abnormalities as described below were generally scored as a single category.

### Microscopy

Light microscopy images of eclosed adult flies were taken with a Nikon Digital DS-Fi1 High-Definition Color Camera, using the Nikon stereoscopic zoom microscope SMZ 745T run by NIS-Elements D 4.20 software. Fluorescence microscopy of flies used a Zeiss Axio Imager 2 microscope (50 × magnification). Z projection images were generated using Carl Zeiss Zen 2012 software.

### Protein analysis by western blotting

Total protein was extracted from batches of 20 pupae crushed in homogenization buffer, and processed as described previously (Andjelković *et al.* 2015). Primary antibodies used were customized rabbit anti-AOX (Fernandez-Ayala *et al.* 2009; 21st Centrury Biochemicals, 1:10,000), and mouse anti-ATP5A (Abcam, 1:100,000), with secondary antibodies as described previously (Andjelković *et al.* 2015).

### Data availability

The authors state that all data necessary for confirming the conclusions presented in the article are represented fully within the article.

## Results

### tubGS plus high levels of RU486 produce developmental abnormalities

**Figure 1:**
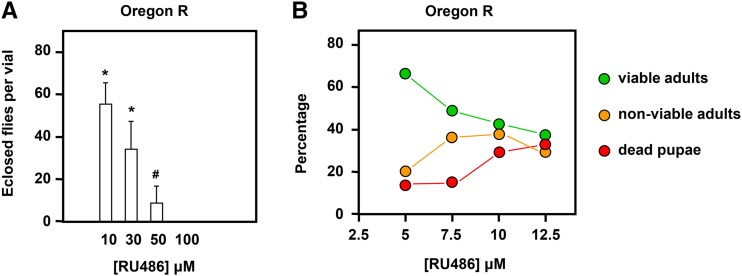
RU486 in combination with *tubGS* produces dose-dependent lethality. (A) Number of *tubGS* progeny eclosing at different doses of RU486 present throughout development, mean ± SD per vial, in OregonR background. Note that at 100 μM, no flies eclosed. * and # indicate significant differences from the next higher concentration tested in pairwise comparisons (Student’s *t*-test, *P* < 0.01 and 0.05, respectively). (B) Proportion (% of pupae formed) of *tubGS* progeny at different doses of RU486 present throughout development; combined data from sets of four vials at a given concentration, set up in parallel, in *w^1118^* background. *n* = 205 (at 5 M), 193 (at 7.5 μM), 210 (at 10 μM), and 146 (at 12.5 μM). *tubGS* plus RU486 produced comparable amounts of pupal lethality also in the CantonS background. SD, standard deviation; *tubGS*; α*-tubulin-GeneSwitch*.

**Figure 2:**
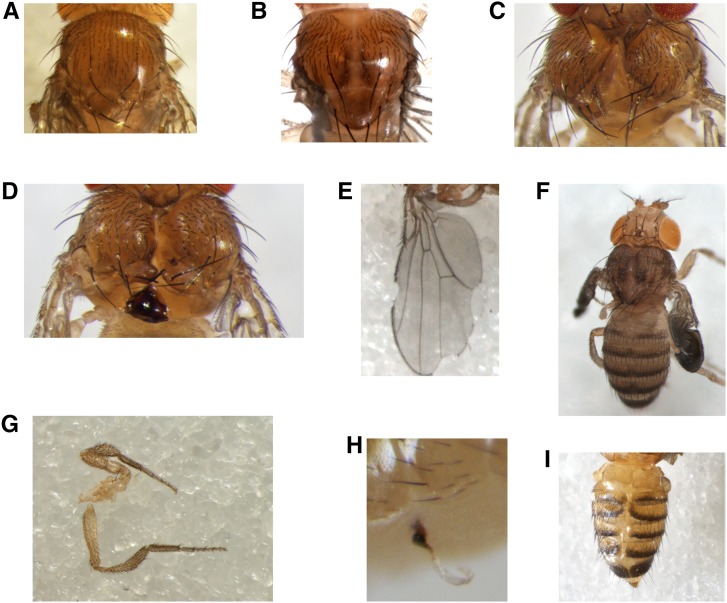
Examples of dysmorphologies produced by the *tubGS* driver in the presence of 10 μM RU486. (A–D) Thoracic abnormalities: (A) missing scutellar part, (B) mild cleft, (C) severe cleft, and (D) necrotic tissue, always localized at the scutellum or notum. (E and F) Wing abnormalities: (E) notched wings, with notches localized on the marginal anterior or posterior side or both, (F) noninflated wings. (G) Leg abnormalities, including overgrown, reduced, and fused leg segments, sometimes present all together. (H) Externalized trachea, always in the ventral abdomen. (I) Abdominal clefting: strong midline splits between all dorsal tergite plates; laterotergites do not fuse at the dorsal midline and remain as hemitergites, with incomplete fusion of abdominal epidermis. These phenotypes were seen in all genetic backgrounds tested (OregonR, CantonS, *w^1118^*, and *w^DAH^*). *tubGS*; α*-tubulin-GeneSwitch*.

In initial trials, we noticed that doses of RU486 used routinely to induce UAS-dependent transgene expression in *Drosophila*, in combination with the *tubGS* driver in adult flies (200–500 μM; Kemppainen *et al.* 2014a), were lethal when present throughout development. In order to investigate possible mechanisms of this lethality, we reared flies at RU486 doses intermediate between this lethal level, and levels sufficient to induce full dose-dependent transgene expression, which in larvae was only 1–2 μM. In combination with *tubGS*, RU486 at 100 μM was still lethal (Figure 1A), whereas *tubGS* flies reared without drug, or wild-type flies reared at this concentration of RU486, developed normally. At intermediate drug concentrations (5–50 μM, Figure 1, A and B), we observed dose-dependent semilethality, although many of the eclosing flies were very weak and died within 1 d. In addition, even the viable flies displayed a range of dysmorphic phenotypes, illustrated in Figure 2 and Supplemental Material, Figure S1 and File S1, of which the commonest and most striking were cleft thorax (Figure 2, B–D) and notched wings (Figure 2E). The observed phenotypes were of varying severity. For example, some flies had single or multiple notches at the wing margin (Figure 2E), whereas others had wings that failed to inflate (Figure 2F). Cleft thorax ranged from severe, with the heminota completely separated (Figure 2, C and D), to very mild, showing only an abnormal, parted bristle pattern or just a reduced scutellum (Figure 2A). A minority of flies also showed necrotic tissue in the notum area (Figure 2D), leg abnormalities such as overgrown, reduced, and fused leg segments (Figure 2G), externalized trachea (Figure 2H), clefted abdomen (Figure 2I), or a variety of malformations of macrochaetae (supernumerary, missing, kinked, or short bristles, Figure S1). Clefting also extended along the abdomen in some cases (Figure 2I). *tubGS* flies reared without drug, or cultured in RU486 in the absence of *tubGS*, did not exhibit cleft thorax or other developmental abnormalities, indicating that these teratogenic effects require the combination of the modified transcription factor plus the inducing steroid.

**Figure 3:**
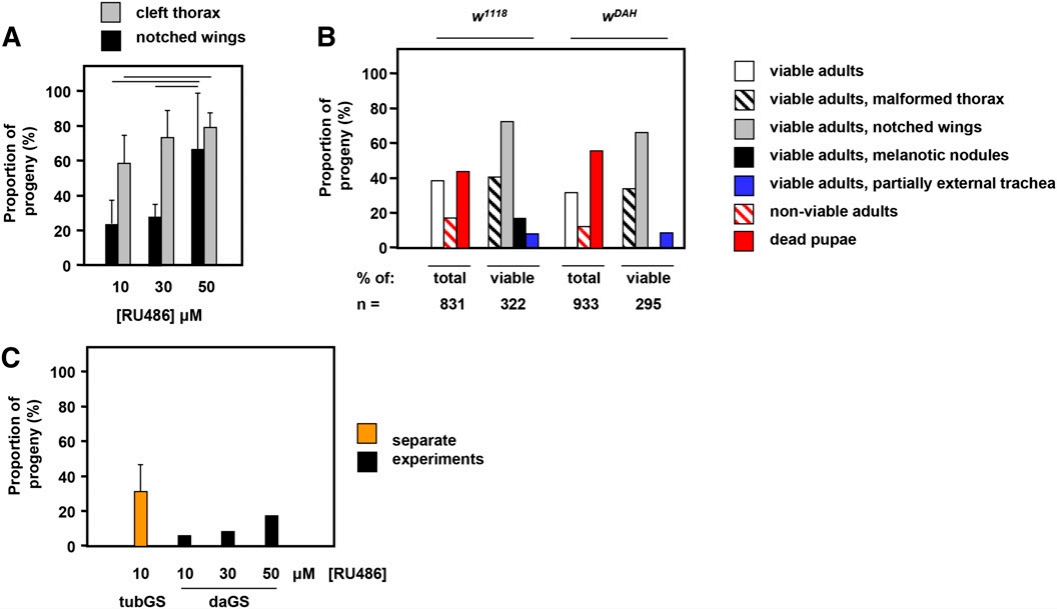
Effects of drug concentration, driver, and genetic background on developmental abnormalities induced by GeneSwitch plus RU486. (A) Proportion of viable adult progeny exhibiting major phenotypic abnormalities as indicated, at different doses of RU486, in the Oregon R genetic background. Mean ± SD for sets of n ≥ 4 independent vials. Horizontal bars denote significant differences for a given phenotypic trait between the stated drug concentrations (Student’s *t*-test, *P* < 0.05). (B) Proportion of progeny in different phenotypic classes of *tubGS* flies in the *w^1118^* and *w^DAH^* backgrounds grown at 10 μM RU486. Note that the adult phenotypes are scored as percentages of the viable adult flies that eclosed. Total numbers of pupae analyzed in each large-scale experiment (n) as indicated. (C) Proportion of adult progeny showing bristle abnormalities, as illustrated in Figure S1, in flies grown at the indicated doses of RU486, bearing the *tubGS* or *daGS* drivers as indicated. Large-scale experiment using the *daGS* driver analyzed *n* = 508 individual adult flies (10 μM), *n* = 758 (30 μM), and *n* = 246 (50 μM). The data for the *tubGS* driver at 10 μM is the mean ± SD for three independent experiments (*n* = 89, 284, and 157 adults analyzed). See also Figure S2. *daGS*, *daughterless-GeneSwitch*; SD, standard deviation; *tubGS*; α*-tubulin-GeneSwitch*.

We quantified the main classes of abnormality and observed a dose-dependence on RU486 (Figure 3A). Although the proportion of progeny showing the two major dysmorphic phenotypes of cleft thorax or notched wings was already substantial at 10 μM RU486, increasing the dose to 30 μM resulted in a significant increase in the proportion exhibiting cleft thorax, whereas a further increase to 50 μM produced a significantly greater proportion with notched wings.

In order to determine whether the induction of these developmental defects was a general property of GeneSwitch drivers, or a phenomenon specific to *tubGS*, we repeated the experiment using a second GeneSwitch driver under the control of the *daughterless* promoter. In contrast to *tubGS*, *daGS* in combination with 10 μM RU486 produced no clefting and no wing defects. The only developmental abnormality detected was in regard to bristle morphology and organization which, while less frequently observed than with the *tubGS* driver, did show a tendency to rise in frequency as the concentration of RU486 was increased (Figure 3C). However, neither cleft thorax nor notched wings were seen at these elevated drug concentrations, nor even at 100 μM. The difference in the findings between the two drivers is most likely attributable to the level and pattern of expression of the GeneSwitch transcription factor, as reflected in its ability to drive transgene expression, which we profiled quantitatively by western blotting using a *UAS-AOX* reporter (Figure S2A) and spatially using a *UAS-GFP* reporter (Figure S2B). Expression of *UAS-AOX* driven by *daGS* was quantitatively much less than when driven by *tubGS*, even at high RU486 concentrations (Figure S2A). Furthermore, unlike *tubGS*, which was able to drive expression ubiquitously in the developing larva, *daGS* produced transgene expression only in a minority of cells (Figure S2B), including salivary glands, parts of the trachea, some epithelial cells, and segmentally reiterated cell clusters.

### Expression of AOX, but not Ndi1 or GFP, rescues cleft thorax caused by tubGS/RU486

**Figure 4:**
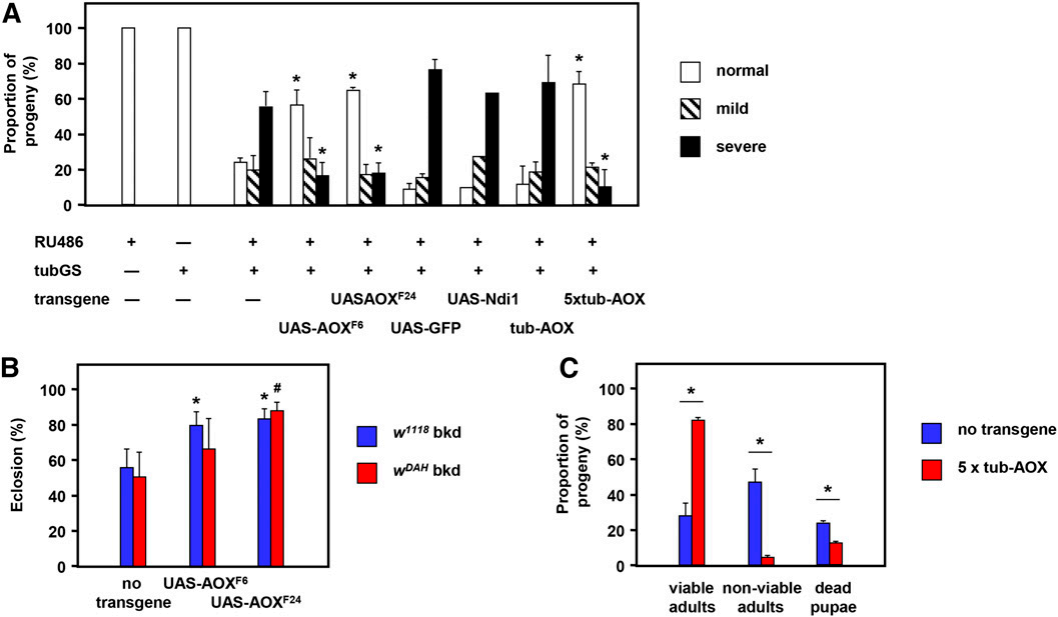
AOX partially rescues cleft thorax and developmental lethality of *tubGS*/RU486. Proportion of adult progeny exhibiting the indicated phenotypes, with hemizygous transgenes as indicated, cultured with (+) or without (–) 10 μM RU486. n ≥ 3 replicate vials for each genotype studied (except *UAS-Ndi*, *n* = 2, hence no error bars shown). Transgenic lines containing *tub-AOX* transgenes (Kemppainen *et al.* 2014) had either a single hemizygous copy or else five copies (two homozygous, plus hemizygous copy on chromosome 3, combined with *tubGS* on the same chromosome). * denotes data classes significantly different from the equivalent class for control lacking any transgene additional to *tubGS* (Student’s *t*-test with Bonferroni correction, *P* < 0.01). (B) Proportion of pupae from two different genetic backgrounds (bkd), as shown, eclosing after culture in 10 μM RU486. All pupae carried the tubGS driver and either no other transgene, or either of two different *UAS-AOX* transgenes, as indicated. # and * denote data classes significantly different from nontransgenic flies in the same genetic background (Student’s *t*-test, *P* < 0.05 or 0.01, respectively). (C) Proportion of pupae eclosing as viable or nonviable adults after culture in 10 μM RU486. All pupae carried the *tubGS* driver and either no other transgene, or else five copies of tub-AOX transgenes (see above). Nonviable adults were those that died on the day of eclosion. * denotes phenotypic classes of transgenic flies significantly different from corresponding class of nontransgenic flies (Student’s *t*-test, *P* < 0.01). AOX, alternative oxidase; GFP, green fluorescent protein; *tubGS*; α*-tubulin-GeneSwitch*.

We tested whether concomitant expression of other transgenes driven by *tubGS* in the presence of RU486 was able to modify the developmental phenotypes resulting from the driver and drug alone (Figure 4). Once again, neither *tubGS* nor the drug on its own produced cleft thorax (Figure 4A) but, when combined, over 50% of the eclosing progeny manifested severe cleft thorax, and a further 20% showed mild clefting. Coexpression of *Ciona* AOX from either of two *UAS-AOX* transgenic lines (Fernandez-Ayala *et al.* 2009) produced a substantial rescue of the phenotype, with over 50% of the eclosing progeny now showing no cleft, and less than 20% having severe cleft. *UAS-Ndi1* or *UAS-GFP* produced no rescue of the phenotype. Nor did a single copy of AOX, when constitutively expressed under the α*-tubulin* promoter at a much lower level than when driven by *tubGS* (Kemppainen *et al.* 2015). However, five copies of the *tub-AOX* transgene, when present simultaneously, did produce a rescue comparable with that of *UAS-AOX*. Coexpression of *UAS-AOX* with *tubGS* plus drug, in either of two backgrounds commonly used in transgenic studies (*w^1118^* and *w^DAH^*) also increased the proportion of pupae eclosing (Figure 4B). The simultaneous presence of five *tub-AOX* transgenes (Figure 4C) also substantially rescued the eclosion frequency, as well as the survival of adults immediately after eclosion.

### AOX rescues developmental abnormalities in a dose-dependent manner

**Figure 5:**
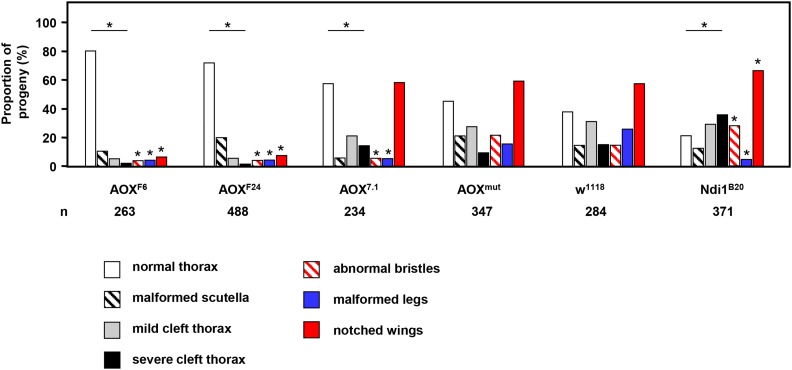
AOX rescues diverse developmental abnormalities produced by *tubGS*. Proportion of progeny hemizygous for both *tubGS* and the indicated transgenes, which exhibited the indicated developmental abnormalities, when reared on food containing 10 μM RU486. The total numbers of flies of each genotype analyzed, in a single large-scale experiment (n), is as shown. See supplemental material for a detailed description of phenotypic categories. Asterisks indicate significant differences (*P* < 0.001) from the *w^1118^* background strain hemizygous for *tubGS*, based on chi-squared analysis for each phenotypic category or for the four thoracic phenotypes (normal thorax, malformed scutellum, mild cleft, and severe cleft) considered as a whole. AOX, alternative oxidase; *tubGS*; α*-tubulin-GeneSwitch*.

We next conducted a large-scale experiment, analyzing almost 2000 individual flies, for each of the major classes of developmental abnormality produced by tubGS in the presence of RU486, in the presence of different UAS-dependent transgenes (Figure 5). As negative control we used strain *w^1118^*, the background strain for all the transgenic lines that were crossed in the experiment. To determine whether the failure of a single copy of *tub-AOX* to rescue *tubGS*-induced cleft thorax was due to low expression, we made use of an additional *UAS-AOX* line, *UAS-AOX^7.1^* (Andjelković *et al.* 2015), showing much lower expression than either of *UAS-AOX^F6^* or *UAS-AOX^F24^*. Finally, to confirm that the enzymatic activity of AOX is required for the rescue, we also included a line (*UAS-AOX^mut^*) expressing a catalytically inactive variant of AOX (Andjelković *et al.* 2015). The proportion of abnormal phenotypes obtained using *UAS-AOX^mut^* was virtually indistinguishable from the background strain *w^1118^*, while the weakly expressing *UAS-AOX^7.1^* transgene produced an intermediate spectrum of phenotypes, with cleft thorax, leg, and bristle abnormalities significantly improved over the background strain, but to a much lower extent than seen with the strongly expressing lines *UAS-AOX^F6^* and *UAS-AOX^F24^*. *UAS-AOX^7.1^* also produced no rescue of the notched wings phenotype, while *UAS-Ndi1^B20^* significantly exacerbated all of the abnormal phenotypes compared with the background strain, with the exception of leg malformations, which were decreased in frequency.

## Discussion

In this study, we identified a range of developmental abnormalities associated with the use of the *tubGS* driver in combination with RU486. These were seen at concentrations only slightly above those commonly used to induce transgene expression in *Drosophila* during development. At concentrations of 2.5 μM or above, we observed substantial pupal lethality, while at 10 μM or above the majority of viable eclosed adults had visible dysmorphic features, commonly including notched wings and cleft thorax. Importantly, these phenotypes were dependent on both the driver and the drug: neither alone produced any evidence of developmental lethality or abnormality, and the effects did not appear to be background dependent, since they were seen in wild-type OregonR and Canton-S flies, as well as in two white-eyed lines commonly used in transgenic studies. A different GeneSwitch driver, with a much lower and more restricted expression pattern, based on its ability to drive GFP expression (Figure S2), produced only very subtle abnormalities in bristle organization.

### Mechanism of developmental disturbance by tubGS/RU486

Previous authors have noted that RU486 treatment alone produces no detectable abnormal phenotypes, although expression of a small number of mRNAs is altered in adults treated with the drug (Etter *et al.* 2005). Given that we also saw no abnormalities from the use of *tubGS* or RU486 on their own, we can exclude the possibility that RU486 binds to or interferes with the activity of known nuclear receptors in *Drosophila* (Fahrbach *et al.* 2012), or that the GeneSwitch transcription factor is able to interact with any of their physiological ligands. However, ligand-bound GeneSwitch may be able to interact either with one or more of these receptors, its targets, or other regulatory factors involved in developmental patterning; for example, by the formation of nonphysiological heterodimers between ligand-bound GeneSwitch and *bona fide* nuclear receptors.

The major dysmorphologies we observed have been reported previously in a variety of mutants, often in combinations similar to those that we observed. Cleft thorax has been reported in mutants of *Ultraspiracle* (Henrich *et al.* 1994), a dimerization partner of the ecdysone receptor and thus one of the key nuclear receptors regulating development progression in the fly. It has also been reported in mutants of the GATA transcription factor *pannier* (Heitzler *et al.* 1996) and the zinc-finger pair-rule transcription factor gene *odd* (Tripura *et al.* 2011). Bristle abnormalities similar to those that we observed are also characteristic of mutants of the dimerization partner of *pannier*, *u-shaped* (Cubadda *et al.* 1997).

Mutants in the components of the AP-1 transcription factor, *jun-related antigen* (homolog of mammalian c-Jun) and *kayak* (homolog of mammalian c-Fos), as well as in the JNK signaling pathway that links AP-1 activity to various upstream developmental signals, cause cleft thorax (reviewed by Zeitlinger and Bohmann 1999; Kockel *et al.* 2001). Defects in JNK signaling also underlie wing defects and leg malformations (Kirchner *et al.* 2007), and have been implicated in midline closure defects in mammals (Chi *et al.* 2005; Zhu *et al.* 2016). Cleft-thorax can result both from downregulation of effectors of JNK signaling, such as the serine protease scarface (Srivastava and Dong 2015), or from mutations in receptor tyrosine kinase Pvr (Garlena *et al.* 2015), an upstream JNK pathway activator (Ishimaru *et al.* 2004; Igaki 2009). Thoracic closure also depends on downstream targets such as proteins implicated in cytokinesis and cell adhesion (Sfregola 2014), as well as intracellular protein trafficking (Thomas *et al.* 2009). Mutants of *blistery*, encoding tensin, result in blistered wings, and interact also with JNK signaling (Lee *et al.* 2003). Overexpression of the inhibitor of matrix metalloproteases (*Timp*) results in pupal lethality and cleft thorax (Srivastava *et al.* 2007). Finally, wing disc-specific knockdown of Tap42, a key regulator of protein phosphatases, gives rise to cleft thorax and to wing abnormalities similar to some that we observed (Wang *et al.* 2012).

Notched wings are another previously observed phenotype in many mutants, including those affecting the highly pleiotropic intercellular signaling factor Notch (originally discovered by Morgan; Welshons 1958), SNARE-dependent membrane trafficking (Stewart *et al.* 2001), protein phosphatase PP2A (Kunttas-Tatli *et al.* 2009), the RNA-binding fragile X protein FMR1 (Wan *et al.* 2000), and histone deacetylation (Pile *et al.* 2001).

The exact pattern of developmental abnormalities brought about by GeneSwitch together with its ligand appears to reflect the tissue specificity of its expression. Thus, whereas the widely expressed *tubGS* produces a plethora of abnormal phenotypes, *daGS*, with much more restricted larval expression (Figure S2), primarily in segmentally reiterated clusters of cells that might represent larval sense organs (Brewster and Bodmer 1995), has only a single visible phenotype in the adult, affecting the sensory bristles (Figure 3C). The use of other GeneSwitch drivers may help to further clarify how its level and pattern of expression affect the phenotypic outcome.

Finding a common thread through this rather bewildering array of phenotypes and genetic pathways may not be straightforward. However, transcriptional cascades are considered to be the main determinants of developmental processes, and the key system for regulating morphogenesis at pupal stage is the steroid hormone 20-hydroxyecdysone (Riddiford 1993). Thus, an interference with ecdysteroid-dependent transcription is the most parsimonious explanation for the pleiotropic effects we observed, even though molecular details remain to be filled in.

### Mechanism of AOX rescue of developmental disturbance by tubGS/RU486

While the observation that GeneSwitch-plus-RU486 can produce a range of developmental abnormalities may be unexpected, their rescue by a mitochondrially localized electron-transfer protein from another phylum is even more surprising. It is important to note that, while the abnormal phenotypes were produced by using an engineered (and thus nonphysiological) transcription factor, and were rescued by a gene from a distant phylum, the effects were systematic in both cases, indicating meaningful underlying biological processes. Thus, the extent of AOX rescue of pupal lethality, cleft thorax, and other dysmorphologies was dependent on the AOX expression level, since strains expressing only at a low level (single-copy of constitutive *tub-AOX*, or low-expressor GAL4-dependent line *UAS-AOX^7.1^*) produced a less dramatic alleviation of the phenotypes studied than the corresponding high-expressors (5 × *tub-AOX*, *UAS-AOXF^24^*, and *UAS-AOX^F6^*). Rescue was dependent on the enzymatic activity of AOX and was not seen with an inert reporter protein (GFP) or a different mitochondrially localized electron-transfer protein, yeast Ndi, which appeared to exacerbate some phenotypes. AOX maintains ATP production, redox homeostasis, and metabolic flux under physiological conditions where respiratory complexes III and IV are limiting due to overload, toxins, or genetic damage, and concomitantly limits mitochondrial ROS production consequent upon overreduction of the quinone pool (El-Khoury *et al.* 2014). AOX also has an unexplained antioxidant effect, decreasing net mitochondrial ROS output even under conditions where the respiratory chain is functioning normally (Fernandez-Ayala *et al.* 2009; Sanz *et al.* 2010a).

How this links to a global alleviation of developmental perturbations brought about by interference with transcriptional cascades or cell signaling is far from clear. In a general sense, our findings hint at a common metabolic regulation of transcription, such as evidenced previously by AMPK sirtuins or PARP (Kraus and Lis 2003; Ghosh *et al.* 2010; Gut and Verdin 2013; Schiewer and Knudsen 2014; Salminen *et al.* 2016), although none of these is obviously implicated, so a novel pathway may be involved. In mice, nuclear receptors are responsive to a variety of metabolic effectors, which can also be microbiome-dependent (Montagner *et al.* 2016), while cross-talk between nutrient-based sensors and nuclear receptors is dependent on mitochondrial stress signals and influences mitochondrial gene expression (Kang *et al.* 2015).

Many transcription factors, including nuclear receptors such as LXRα (Serviddio *et al.* 2013) or NR4A1 (Shimizu *et al.* 2015) in mammals, are known to be activated in response to oxidative stress (Lavrovsky *et al.* 2000), and redox regulation of nuclear receptors such as the glucocorticoid receptor (Tanaka *et al.* 1999) is well established. AOX may therefore act by providing a general dampening of ROS, normalizing developmental outcomes dependent on such receptors, with which GeneSwitch plus RU486 interferes. An exhaustive study using different ROS scavengers may shed further light on this.

Another possibility is based on the observation that synthesis of 20-hydroxyecdysone requires mitochondrial Fe-S cluster-containing proteins dependent on frataxin (Palandri *et al.* 2015) and mitoferrin (Llorens *et al.* 2015). Because Fe-S proteins are highly susceptible to ROS damage, a general ROS dampening effect of AOX may counteract transcriptional interference from ligand-bound GeneSwitch, simply by boosting endogenous ecdysteroid synthesis.

### Recommendations on use of GeneSwitch drivers

The GeneSwitch system was originally elaborated using other drivers than *tubGS*, *i.e.*, those linked to the neuron- and muscle-specific *elav* and *Mhc* promoters, respectively (Osterwalder *et al.* 2001), or for specific expression in other tissues such as the fat body (Roman *et al.* 2001). Subsequently, the “ubiquitous” GS drivers (such as *tubGS* and *Actin5C-GS*) have been brought into use for inducing broad expression, both in adults and larvae (Ford *et al.* 2007; Waskar *et al.* 2009; Wigby *et al.* 2011; Paik *et al.* 2012; Kuo *et al.* 2012; Kemppainen *et al.* 2014a,b; Sun *et al.* 2014; Da-Rè *et al.* 2014).

Our work raises at least two concerns. First, the visible interference with developmental processes at saturating or near-saturating drug concentrations, using the *tubGS* driver, indicates the need for rigorous controls and cautious interpretation of all data obtained using this driver during development. Furthermore, we obviously cannot rule out subtler but also biologically significant effects that did not have visible manifestations, even at lower drug concentrations than those employed here. Second, other GeneSwitch drivers activated during development may also be vulnerable to such effects, since our data indicate that they depend on the drug and the transcription factor in combination, which applies wherever they are colocated. An example would be the recently published use of a GeneSwitch driver to overexpress malic enzyme (Kim *et al.* 2015). The driver in this example was originally reported to induce expression in the adult abdominal fat body (Hwangbo *et al.* 2004), although Kim *et al.* (2015) found that expression in larvae was instead driven in the salivary glands, Malpighian tubule, and part of the gut. In this particular paper, the appropriate controls without the transgene were indeed implemented for the adult (see Supplementary Table 1C of Kim *et al.* 2015), but some questions remain. The driver plus drug alone did not affect the body weight of L3 larvae (Figure 3A of Kim *et al.* 2015), but effects on stress resistance and lifespan in such controls were not documented. The concentrations of RU486 used by Kim *et al.* (2015), *i.e.*, 2.5–10 μg/ml, corresponding with 5.8–23 μM, were within the range in which we saw major developmental effects using the *tubGS* driver. Similarly, in flies expressing GeneSwitch in specific endocrine cells during development, using a customized driver and RU486 at even higher concentrations from larval L2 stage onwards (Cho *et al.* 2014), clear developmental abnormalities were attributed to knockdown of a nuclear receptor, although driver-plus-drug controls were not included in all of the experiments reported. Some phenotypes observed (Figure 3 of Cho *et al.* 2014) resemble those that we report here (pupal lethality, uninflated wings, abdominal clefting, and leg malformations). While their interpretation that these are due to disrupted ecdysone signaling may be correct, an effect of GeneSwitch plus RU486 in the target cells cannot be excluded. Phenotypic rescue by injected ETH (Table 2 of Cho *et al.* 2014) confirmed the involvement of disrupted ecdysis, but not the underlying causes thereof. A further possible example already reported in the literature is the effect of the abdominal fat body-specific GeneSwitch driver on lifespan, when RU486-containing food was supplied in the adult to drive the supposedly inert GFP transgene (Ren and Hughes, 2014. RU486-dependent lethality in larvae containing the Elav-GeneSwitch driver (Shen *et al.* 2009), and embryonic lethality produced by either the Elav- or Actin5C-GeneSwitch drivers plus maternal RU486 (Landis *et al.* 2015), have been previously reported.

We would recommend that future users of all GeneSwitch drivers should routinely include otherwise nontransgenic controls bearing the drivers, plus and minus drug, in all experiments. Based on our findings (Figure S2B), the *daGS* driver is clearly not ubiquitous, despite the fact that the *daughterless* gene itself, as well as the “standard” *daGAL4* drivers, do show widespread expression.

## Supplementary Material

Supplemental Material
