# Supplementary material for: Ligand-Bound GeneSwitch Causes Developmental Aberrations in Drosophila that Are Alleviated by the Alternative Oxidase
Source: G3 (Bethesda). 2016 Jul 12;6(9):2839–46. doi: 10.1534/g3.116.030882 (PMC5015941; doi:10.1534/g3.116.030882)
Supplement: Supplemental Material [file supp_6_9_2839__index.html]

Ligand-Bound GeneSwitch Causes Developmental Aberrations in Drosophila that Are Alleviated by the Alternative Oxidase — Supplemental Material 

# Ligand-Bound GeneSwitch Causes Developmental Aberrations in *Drosophila* that Are Alleviated by the Alternative Oxidase

## Supplemental Material for Andjelković, Kemppainen, and Jacobs *et al*, 2016

**Files in this Data Supplement:**

- File S1 - This file contains both supplemental figures and their corresponding legends. (.pdf, 403 KB)
- Figure S1 - Examples of sensory bristle defects on the notum, produced by GeneSwitch drivers in presence of RU486. (.ppt, 7.65 MB)
- Figure S2 - Profiling of expression driven by the *tubGS* and *daGS* drivers. (.ppt, 6.86 MB)
