# Supplementary material for: Ligand-Bound GeneSwitch Causes Developmental Aberrations in Drosophila that Are Alleviated by the Alternative Oxidase
Source: G3 (Bethesda). 2016 Jul 12;6(9):2839–46. doi: 10.1534/g3.116.030882 (PMC5015941; doi:10.1534/g3.116.030882)
Supplement: Supplemental Material [file supp_g3.116.030882_FileS1.pdf]

Ligand-bound GeneSwitch

causes developmental aberrations in *Drosophila* that

are alleviated by the alternative oxidase

Ana Andjelković, Kia K. Kemppainen,

& Howard T. Jacobs

SUPPLEMENTARY DATA

LEGENDS TO SUPPLEMENTARY FIGURES

Figure S1

Examples of sensory bristle defects on the notum, produced by GeneSwitch drivers in presence of RU486

Wild type flies have four scutellar bristles at the notum. (A) Extra (ectopic, six) scutellar macrochaetes. (B) Missing (three) scutellar macrochaetes. (C) One of the scutellar macrochaetes is shorter and missoriented (red arrow); one of the notum macrochaetes appears bent (white arrow). (D) Bent scutellar macrochaete.

Figure S2

Profiling of expression driven by the *tubGS* and *daGS* drivers

(A) Western blot of protein extracts from pupae expressing AOX (line *UAS-AOX*^{F6}), driven by the indicated drivers and RU486 concentrations, and probed for AOX and for ATPα as loading control. (B) Apotomized fluorescent micrographs of L3 larvae expressing nucleus-localized GFP (line *UAS-GFP* Stinger), driven by the indicated drivers in presence of 10 mM RU48. Scale bars 0.5 mm. In other images, *daGS* clearly produed low-level expression of GFP also in parts of the trachea and some epithelial cells as well as the salivary glands and segmentally reiterated cell clusters most obvious in the image shown.

A

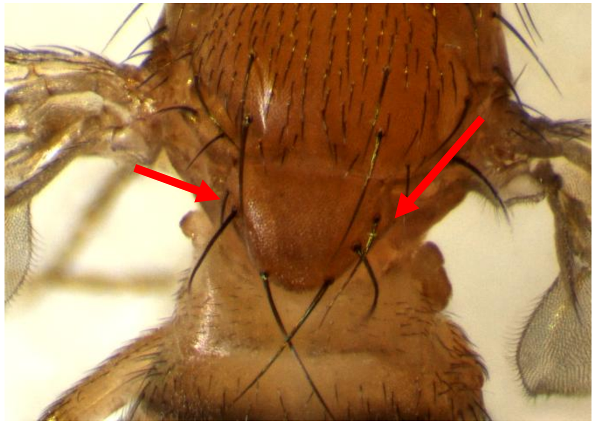

extra

B

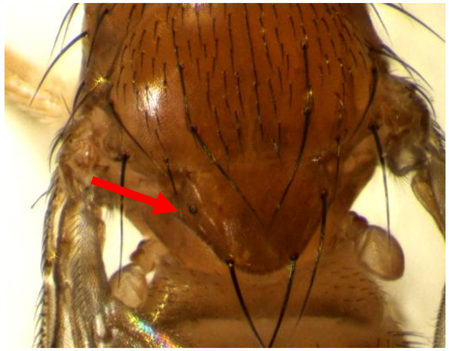

missing

C

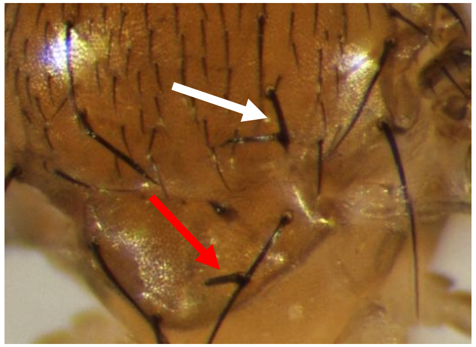

broken or short

D

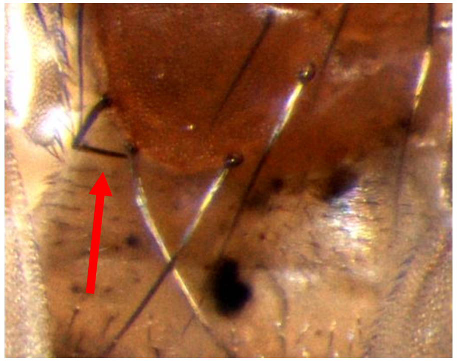

kinked

Andjelković et al, Figure S1

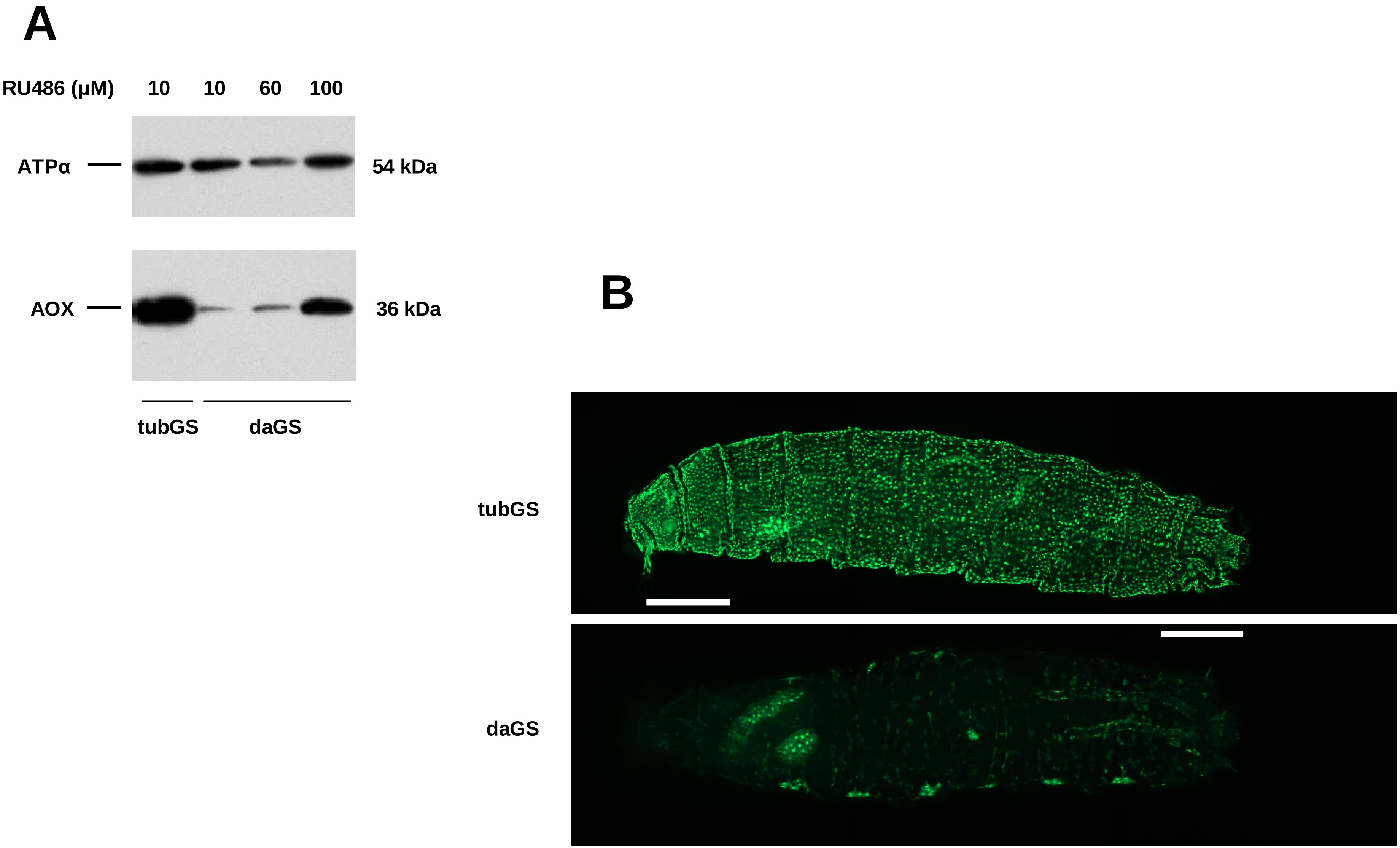

Andjelković et al, Figure S2
