# Supplementary figures and images for: Ligand-Bound GeneSwitch Causes Developmental Aberrations in Drosophila that Are Alleviated by the Alternative Oxidase
Source: G3 (Bethesda). 2016 Jul 12;6(9):2839–46. doi: 10.1534/g3.116.030882 (PMC5015941; doi:10.1534/g3.116.030882)

## Slide 1
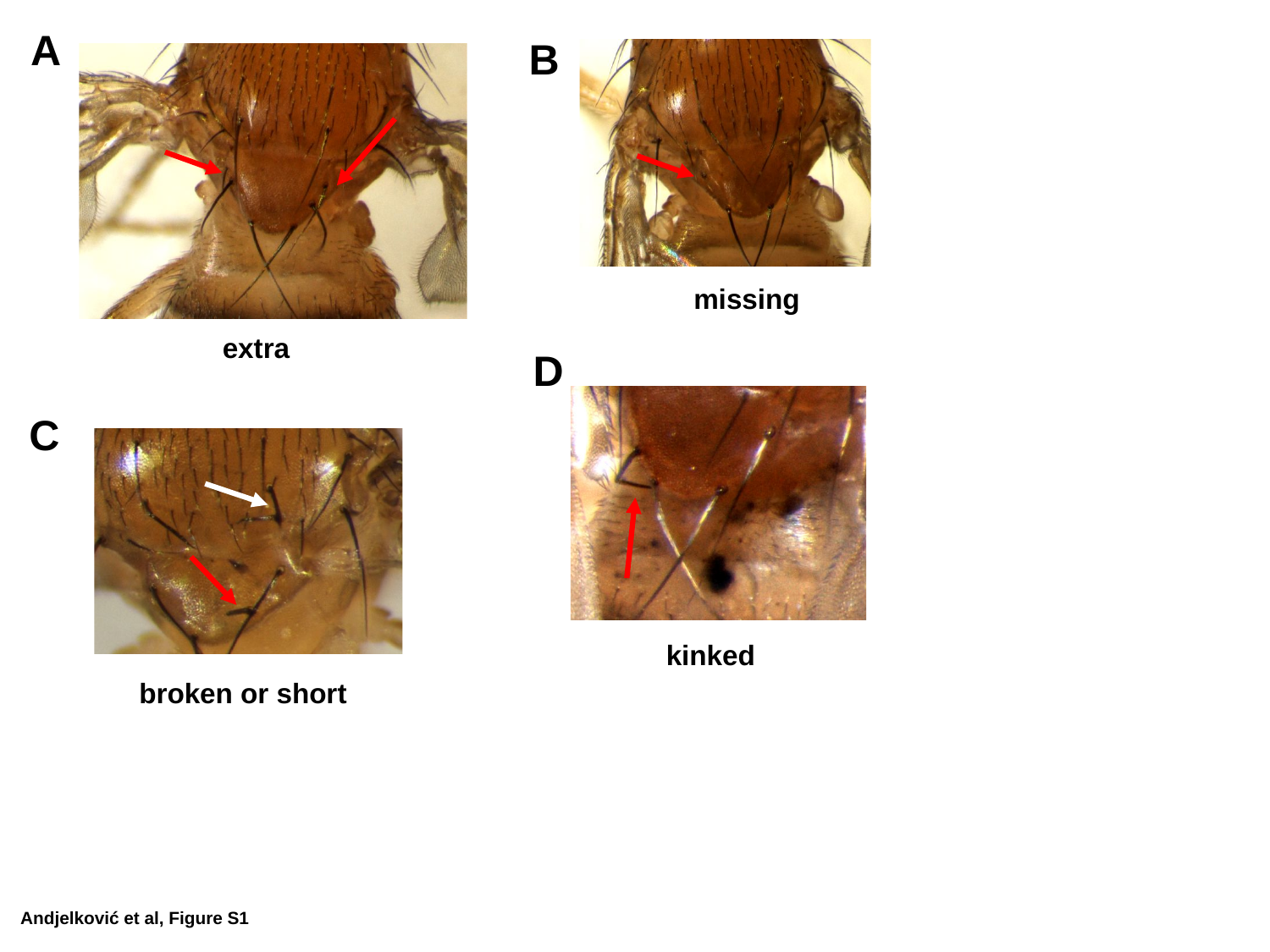

A
B
missing
extra
D
C
kinked
broken or short
Andjelković et al, Figure S1

Supplement: Supplemental Material [file supp_g3.116.030882_FigureS1.ppt]

## Slide 1
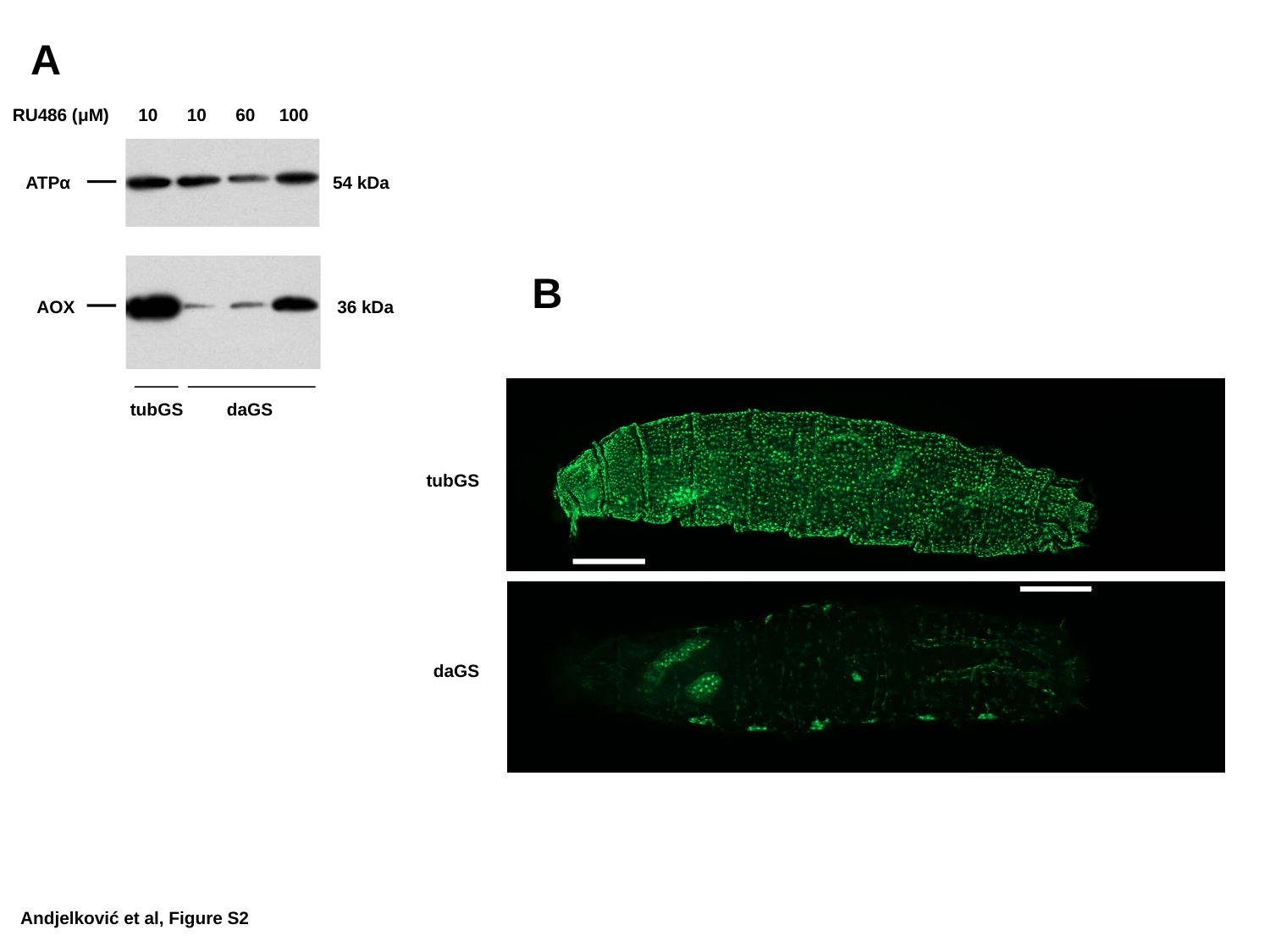

A
RU486 (μM) 10 10 60 100
ATPα 54 kDa
B
AOX 36 kDa
tubGS daGS
tubGS
 daGS
Andjelković et al, Figure S2

Supplement: Supplemental Material [file supp_g3.116.030882_FigureS2.ppt]
